# Investigating data-driven biological subtypes of psychiatric disorders using specification-curve analysis

**DOI:** 10.1017/S0033291720002846

**Published:** 2022-04

**Authors:** Lian Beijers, Hanna M. van Loo, Jan-Willem Romeijn, Femke Lamers, Robert A. Schoevers, Klaas J. Wardenaar

**Affiliations:** 1 Department of Psychiatry, University of Groningen, University Medical Center Groningen, Interdisciplinary Center Psychopathology and Emotion regulation (ICPE), Groningen, The Netherlands; 2 Faculty of Philosophy, University of Groningen, Groningen, The Netherlands; 3 GGZ inGeest and Department of Psychiatry, Amsterdam Public Health Research Institute, VU University Medical Center, Amsterdam, The Netherlands; 4 Department of Psychiatry, University of Groningen, University Medical Center Groningen, Research School of Behavioural and Cognitive Neurosciences, Groningen, The Netherlands

**Keywords:** biochemistry, cluster analysis, complexity, heterogeneity, psychiatry, specification-curve analysis, subtyping

## Abstract

**Background:**

Cluster analyses have become popular tools for data-driven classification in biological psychiatric research. However, these analyses are known to be sensitive to the chosen methods and/or modelling options, which may hamper generalizability and replicability of findings. To gain more insight into this problem, we used Specification-Curve Analysis (SCA) to investigate the influence of methodological variation on biomarker-based cluster-analysis results.

**Methods:**

Proteomics data (31 biomarkers) were used from patients (*n* = 688) and healthy controls (*n* = 426) in the Netherlands Study of Depression and Anxiety. In SCAs, consistency of results was evaluated across 1200 k-means and hierarchical clustering analyses, each with a unique combination of the clustering algorithm, fit-index, and distance metric. Next, SCAs were run in simulated datasets with varying cluster numbers and noise/outlier levels to evaluate the effect of data properties on SCA outcomes.

**Results:**

The real data SCA showed no robust patterns of biological clustering in either the MDD or a combined MDD/healthy dataset. The simulation results showed that the correct number of clusters could be identified quite consistently across the 1200 model specifications, but that correct cluster identification became harder when the number of clusters and noise levels increased.

**Conclusion:**

SCA can provide useful insights into the presence of clusters in biomarker data. However, SCA is likely to show inconsistent results in real-world biomarker datasets that are complex and contain considerable levels of noise. Here, the number and nature of the observed clusters may depend strongly on the chosen model-specification, precluding conclusions about the existence of biological clusters among psychiatric patients.

## Introduction

Heterogeneity is a key feature of almost all psychiatric disorders (Kapur, Phillips, & Insel, 2012; Kendell & Jablensky, 2003). Psychiatric patients usually present with a wide variety of clinical features [e.g. symptom patterns or treatment response (Georgiades, Szatmari, & Boyle, 2013; Kofler et al., 2017; Monroe & Anderson, 2015; Picardi et al., 2012; Volavka & Citrome, 2009)], and different underlying biological disturbances could be at play for patients with the same diagnosis (Ozomaro, Wahlestedt, & Nemeroff, 2013). Identification of more homogeneous diagnostic (sub)groups within larger diagnostic groups (e.g. depression, developmental disorders, psychosis) is often proposed as a starting point for increasing our understanding of more patient-specific etiological mechanisms, and thus, to advance the development of more biologically-informed, patient-specific diagnoses, and personalized treatment (e Silva, 2013; Kapur et al., 2012; Ozomaro et al., 2013).

Identification of psychiatric diagnoses and subtypes has traditionally been based on clinical judgement and consensus (Kendler, 2009). Data-driven cluster analyses can be used to further reduce psychopathological heterogeneity by identifying patterns in data that are missed by clinical pattern recognition (Marquand, Wolfers, Mennes, Buitelaar, & Beckmann, 2016). Although the call to apply data-driven approaches to psychiatric disease classification has been around for decades (Kendell, 1989), their popularity rose notably in recent years (Beijers, Wardenaar, van Loo, & Schoevers, 2019b; Librenza-Garcia et al., 2017; Lombardo, Lai, & Baron-Cohen, 2019; Marquand et al., 2016; Schnack, 2017; Van Loo, De Jonge, Romeijn, Kessler, & Schoevers, 2012). This is likely due to a combination of factors, including the availability of suitable datasets, increased computational capabilities and ongoing advances in the fields of statistics and machine learning that make it possible to extract information from complex and high-dimensional data (Ahmad & Fröhlich, 2016; Lin & Hsien-Yuan, 2017; Marquand et al., 2016). Data-driven clustering techniques have been used to gather evidence about possible subtypes in a broad range of psychiatric patient populations, including depression (Beijers et al., 2019b; Van Loo et al., 2012), psychosis (Chand et al., 2019; Lewandowski, Baker, McCarthy, Norris, & Öngür, 2018; Reser, Allott, Killackey, Farhall, & Cotton, 2015), bipolar disorder (Librenza-Garcia et al., 2017) and developmental disorders (e.g. attention deficit hyperactivity disorder (Mostert et al., 2018), autism spectrum disorder (Lombardo et al., 2019)).

The predominant approach used in psychiatry has been unsupervised learning in the form of finite mixture models (FMMs) and clustering algorithms (i.e. k-means clustering, hierarchical clustering, and community detection) (Marquand et al., 2016). Unsupervised methods have been widely used for discovering subtypes within clinical groups because supervised learning, which aims to correctly predict the subject labels (e.g. patients *v.* healthy control), is fundamentally limited by the quality of the clinical labels and cannot be used to investigate the validity of these labels (Wolfers, Buitelaar, Beckmann, Franke, & Marquand, 2015). Unsupervised learning does not use labels but rather attempts to find subgroups based on data structure and heuristics used by each algorithm. Although the use of data-driven clustering techniques seems promising, there is also a reason for caution. Scientific results are known to not always be robust and specifics of a chosen analytical method can have a significant influence on research outcomes (Simmons, Nelson, & Simonsohn, 2011; Steegen, Tuerlinckx, Gelman, & Vanpaemel, 2016; Silberzahn et al., 2018). In case of cluster analyses, however, there is usually no way of knowing if the results of a presented analysis would have been the same if different model specifications had been used, as researchers will generally perform only one or two separate analyses (Marquand et al., 2016). Better insight into the effects of model specifications on unsupervised clustering results could greatly improve our understanding of data-driven psychiatric subtyping. In addition, it could provide leads for data-driven subtypes of MDD by identification of patterns that are robust to methodological variation.

In unsupervised learning, analytical variations across studies are a realistic risk because of the large availability of different model specifications for unsupervised learning algorithms. This is likely due to the lack of a straightforward way to judge the quality of unsupervised learning results because there is no outcome measure, as opposed to supervised learning, which either succeeds at predicting a predefined outcome or not (Hastie, Tibshirani, & Friedman, 2011). We decided to focus on k-means and hierarchical clustering because these have been shown to be the most commonly used methods across disorders (Marquand et al., 2016) and FMMs have previously been shown to have a number of issues (Borsboom et al., 2016; Hagenaars, 1988; van Loo, Wanders, Wardenaar, & Fried, 2016). Within k-means/hierarchical clustering, there are three main aspects of the method that can vary: (1) algorithm, (2) distance metric (used to determine dissimilarity between data points) and (3) fit index (decides which is the optimal number of clusters). When investigating the 13 studies mentioned by Marquand et al. (2016), we found that *k*-means clustering was used most often, but that a specific rationale or justification for this choice was generally not given (8/13). This is likely due to the fact that because of the aforementioned lack of gold standard, we rely on simulation studies for algorithms (Clifford, Wessely, Pendurthi, & Emes, 2011; Ferreira & Hitchcock, 2009; Hands & Everitt, 1987; Saraçli, Doǧan, & Doǧan, 2013) as well as distances (Clifford et al., 2011; Saraçli et al., 2013) and fit indices (Islam, Alizadeh, van den Heuvel, & GROUP investigators, 2015; Milligan & Cooper, 1985). These studies are performed only rarely and generally have mixed results (Clifford et al., 2011; Ferreira & Hitchcock, 2009; Hands & Everitt, 1987; Islam et al., 2015; Milligan & Cooper, 1985; Saraçli et al., 2013).

The current study aimed to identify clusters in a psychiatric sample and to gain insight into the effects of different model specifications on the results by applying a *Specification-Curve Analysis* [SCA (Simonsohn, Simmons, & Nelson, 2020)] to a selected group of unsupervised machine learning algorithms (k-means clustering and six hierarchical clustering algorithms). SCA was developed to investigate the effects of methodological variations on regression results in psychology but can be also applied to study the effect of different model specifications in unsupervised machine learning analyses. When applied to the current case of cluster analysis, SCA considers the results of a large range of model specifications jointly, instead of using cluster analysis with just one or two model specifications. Because SCA has never been applied to cluster analysis before, we also investigated the influence of data properties such as the true number of existing clusters in the data and varying levels of noise on the SCA outcomes.

For this study, we focused on the identification of biological proteomics-based subtypes of MDD. There have been increasing efforts to identify homogeneous clusters of MDD patients, mainly based on clinical data. The results of these studies tend to be unstable or find subtypes mainly based on severity (Van Loo et al., 2012). Fewer efforts have been based on biological measures (Beijers et al., 2019b). There are some indications that biology-based clustering suffers from a similar degree of variation, likely due (at least in part) to the large variability in used methodology (Beijers et al., 2019b). In this study, we investigated if proteomic-based subtypes are indeed sensitive to different model specifications, or that we could find robust subtypes using proteomics data. Our specific aims were to (1) evaluate the influence of model specifications on the number of identified data-driven biological clusters in MDD, (2) to investigate if SCA identifies clusters with distinct biological patterns that are robust to variations in model specifications, and (3) to run simulations to investigate how data properties influence SCA cluster results.

## Methods and materials

**Fig. 1.**
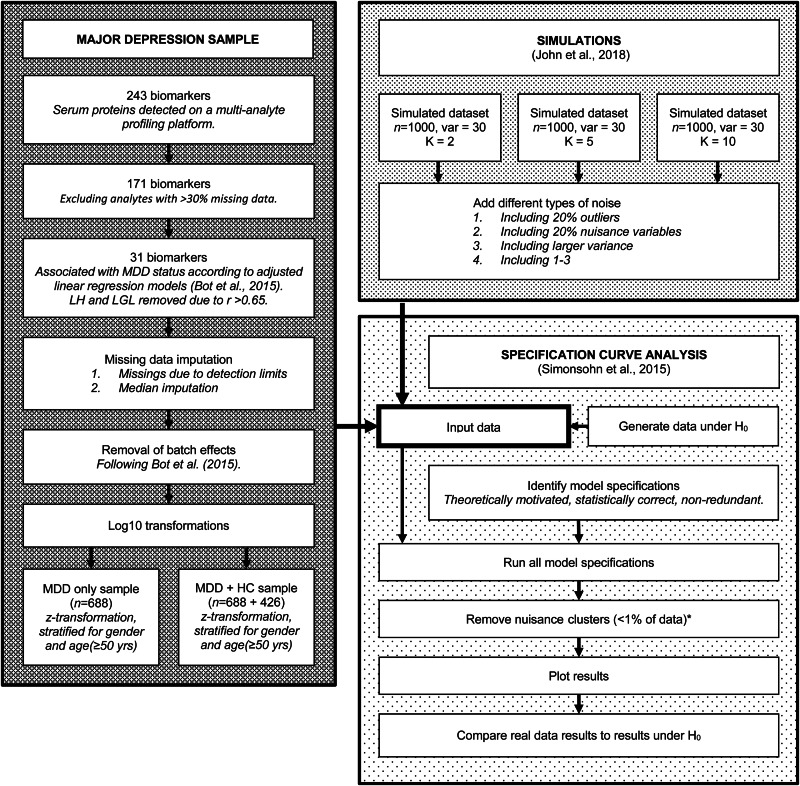
Flowchart of the complete analytical process, including real data preparation, data simulation and specification curve analysis.

For a visual overview of the complete analytical process, see Fig. 1.

### Depression data

#### Participants and procedures

NESDA is a multisite naturalistic cohort study that examines the long-term course of depressive and anxiety disorders. A detailed description of the NESDA design can be found elsewhere (Penninx et al., 2008). In brief, the NESDA cohort consists of 2981 subjects aged 18–65 years, including those with lifetime anxiety and/or depressive disorder and a subgroup of healthy controls. The research protocol was approved by the Medical Ethical Committees of participating institutes, and after a complete description of the study, all respondents provided written informed consent. For the present study, all 688 subjects with a current (past 6 months) diagnosis of MDD according to the Composite International Diagnostic Interview (CIDI; WHO version 2.1) as well as 426 healthy controls were selected. The SCA was first run in the MDD patient sample and then repeated in the combined MDD and healthy control sample (see below).

#### Measurements

Extensive information was gathered through face-to-face interviews, a medical examination, a cognitive computer task and collection of blood samples (Penninx et al., 2008). DSM-IV diagnoses of depressive (minor depression, dysthymia and MDD) and anxiety disorders (Generalized Anxiety Disorder, Social Phobia, Agoraphobia and Panic Disorder) were established using the CIDI. Those without any diagnosis according to the CIDI were included as healthy controls.

#### Proteomic analytes

Blood was sampled after an overnight fast in five research centers throughout the Netherlands (Amsterdam, Leiden, Groningen, Emmen and Heerenveen) and stored at −80 °C. All samples were shipped on dry ice and processed from frozen in a Clinical Laboratory Improvement Amendments-certified laboratory (Myriad RBM; Austin, TX, USA), where a panel of 243 analytes (Myriad RBM DiscoveryMAP 250+) involved in various hormonal, immunological and metabolic pathways were assessed in serum using multiplexed microbead immunoassays. Each batch also contained three duplicate control samples with different protein concentrations, giving an average inter- and intra-assay variability of 10.6% (range 5.5–32.5%) and 5.6% (range 2.5–15.8%), respectively.

#### Analyte data selection

To reduce the likelihood that identified clusters would merely reflect degrees of general somatic health rather than psychopathology (Beijers et al., 2019a), only biomarkers were included that were previously shown to differ between current MDD patients and healthy controls (Bot et al., 2015). We excluded the luteinizing hormone and lactoylglutathione lyase because of correlations >0.65 with follicle-stimulating hormone and macrophage migration inhibitory factor, respectively. A total of 31 biomarkers related to immune response, protein metabolism and diverse cell communication and signal transduction processes were included in the study (See Table 1 and online Supplementary Table S1). Because biomarkers were selected based on their ability to discriminate between MDD and healthy controls, the combined MDD and healthy sample was expected to contain at least two clusters (*K* ⩾ 2).

**Table 1.** Biochemical analytes and associated biological processes

Analyte	Biological process^a^
Alpha-1-antichymotrypsin	PM
Alpha-1-antitrypsin	PM
CD40 antigen	CC,ST
Complement factor h-related protein 1	IM
Enrage	CC,ST
Growth-regulated alpha protein	IM
Interleukin-12P40	IM
Interleukin-1 receptor antagonist	CC,ST
Macrophage migration inhibitory factor	CC,ST
Lactoylglutathione lyase (not included because of high correlation with MIF)	M
Insulin growth factor-binding proteiN-5	CC,ST
Urokinase-type plasminogen activator receptor	CC,ST
Cathepsin D	PM
Receptor tyrosine-protein kinase ERBB-3	CC,ST
Hepsin	PL
Cellular fibronectin	CG
Matrix metalloproteinase-10	PM
Matrix metalloproteinase-3	PM
Tenascin C	CC,ST
Carcinoembryonic antigen	IM
Angiogenin	M
Angiopoietin 2	CC,ST
Vascular endothelial growth factor	CC,ST
Apolipoprotein A4	T
Apolipoprotein D	T
Fatty acid-binding protein, adipocyte	CC,ST
Pancreatic polypeptide	CC,ST
Von willebrand factor	PM
Luteinizing hormone (not included because of high correlation with FSH)	CC,ST
Follicle-stimulating hormone	CC,ST
Cystatin C	PM
Fetuin-A	CC,ST
Prostasin	PM

CC, cell-cell communication; CG, cell growth/maintenance; IM, immune response; M, metabolism; PL, proteolysis and peptidolysis; PM, protein metabolism; ST, signal transduction; T, transport.

a From the Human Protein Reference Database, according to Bot et al. (2015).

#### Data processing

Missing values due to biomarker values being below or above the detection limits were imputed with the values of the lower and upper detection limit, respectively. Other missing values were imputed by the median value (see online Supplementary Table S1 for missing value percentages). We applied the ComBat function (Johnson, Li, & Rabinovic, 2007), including all covariates used previously by Bot et al. (2015), to remove any potential plate effects. Data were log10-transformed to normalize the variance distributions. Because various clustering techniques are sensitive to the relative scaling of variables, we performed z-score transformations, separately for the MDD sample and the combined patient and control sample. Transformations were stratified for gender and age (⩾50 years *v.* <50 years) to prevent these variables from driving the model solutions.

### Specification curve analysis

SCA consists of three steps (Simonsohn et al., 2020). First, the researcher identifies a set of theoretically justified, statistically correct, and non-redundant analytic specifications. Second, the analysis is run with each specification and the results (i.e. number of identified clusters; *y*-axis) are plotted as a function of analysis specification (*x*-axis), which allows for the identification of (in)consistency across specifications. Third, the researcher determines whether the resulting curve is inconsistent with the null hypothesis (H_0_: no clusters present). It is difficult to test the results of any SCA with a statistical test because the specifications are neither statistically independent nor part of a single model (Simonsohn et al., 2020). Therefore, this is done by bootstrapping. The researcher generates many datasets that are in accordance with the null hypothesis (i.e. no clusters present) and runs the complete set of specifications on each of these H_0_ datasets. If the curve based on the real dataset falls outside of the range of expected results based on the bootstrapped H_0_ datasets, H_0_ can be rejected.

#### Analytic specifications

Using the package NbClust_3.0 (Charrad, Ghazzali, Boiteau, & Niknafs, 2014) in R_3.6.1, we performed an SCA with 1200 individual cluster analyses representing all possible model specifications within the most popular non-parametric clustering algorithms [i.e. agglomerative hierarchical clustering and k-means cluster analysis (Jain, 2010)]. Each of the 1200 specifications (see online Supplementary Table S2) represented a unique combination of a *clustering algorithm* (7 options), *distance metric* (determines the distance between data points; 6 options) and *fit index* (identifies the optimal number of clusters; 21 options). Graphical or computationally expensive fit indices were not included. The current large range of available options was included, because there is currently very little evidence to prefer one over the other (Islam et al., 2015).

#### Model selection

In order to approximate what researchers would do when conducting a cluster analysis, we tested 1–15 clusters in each of the 1200 cluster analyses and then selected the best model based on the fit index. In addition, we excluded small clusters (⩽1% of subjects), whilst retaining the other clusters in each model, because small clusters usually include only one or two subjects with extreme values (outliers) and the other clusters may still hold interesting information.

#### Evaluating the null

In order to generate datasets that were in accord with the null hypothesis (H_0_: no clusters present), we created 500 datasets, in which all variables were statistically independent. This was done by selecting a random value from every biological variable for each participant. Next, we ran the SCA in each of these datasets and created the range of expected results. First, the results based on every dataset were ordered from smallest to the largest number of clusters (*K*). Then we combined the 500 results, and the 2.5^th^ and 97.5^th^ percentile for each position 1–1200 were identified, representing the lower and upper of the expected number of clusters (*K*) under H_0_. Therefore, these results do not give the expected range of the specific combination of options, but rather the range of the *m*^th^ smallest *K*. In order for a real-data SCA to reject H_0_, the results must fall outside this range.

#### Cluster stability

Between models with the same *K*, the cluster sizes and allocation of subjects can differ. If *K* clusters truly exist in the data, we expect the model solutions to be relatively stable with respect to these characteristics across different model specifications that yielded *K* clusters. Cluster stability was assessed with a few simple metrics. First, we identified the number of unique model solutions for each group of models with the same number of clusters (*K*). Second, we ranked the models based on the number of times they occurred. Third, we checked the number of solutions that occurred only once in the group of models with the same *K*. Finally, we assessed the stability of subject allocation to clusters by comparing the most often occurring model with the second and third ranking model solutions. We then quantified the number of subjects that switched classes between these model solutions.

### Simulations

We performed a simulation study, aiming to investigate if a known cluster structure is indeed detected as the most consistent in an SCA, and to evaluate the effects of noise and outliers. We simulated datasets using the R-package clusterlab_0.0.2.6 (John et al., 2020). Data were simulated with 2, 5 and 10 clusters, with subjects equally distributed across clusters (total *n* = 1000). The data were simulated with Gaussian variance 1 and circle circumference *K* + 1 to create data without cluster overlap (baseline data). In addition, we simulated noisy datasets with different characteristics:

- Including 20% outliers (distance 4)
- Including 20% nuisance variables (randomly selected values with the same mean/s.d. as the other variables)
- Including a larger variance (*v* *=* 2), in order to have ~30% overlap
- Including all of the above

For the first and second principal component coordinates of these datasets, see online Supplementary Fig. S1.

## Results

### Specification curve analysis in MDD sample

**Fig. 2.**
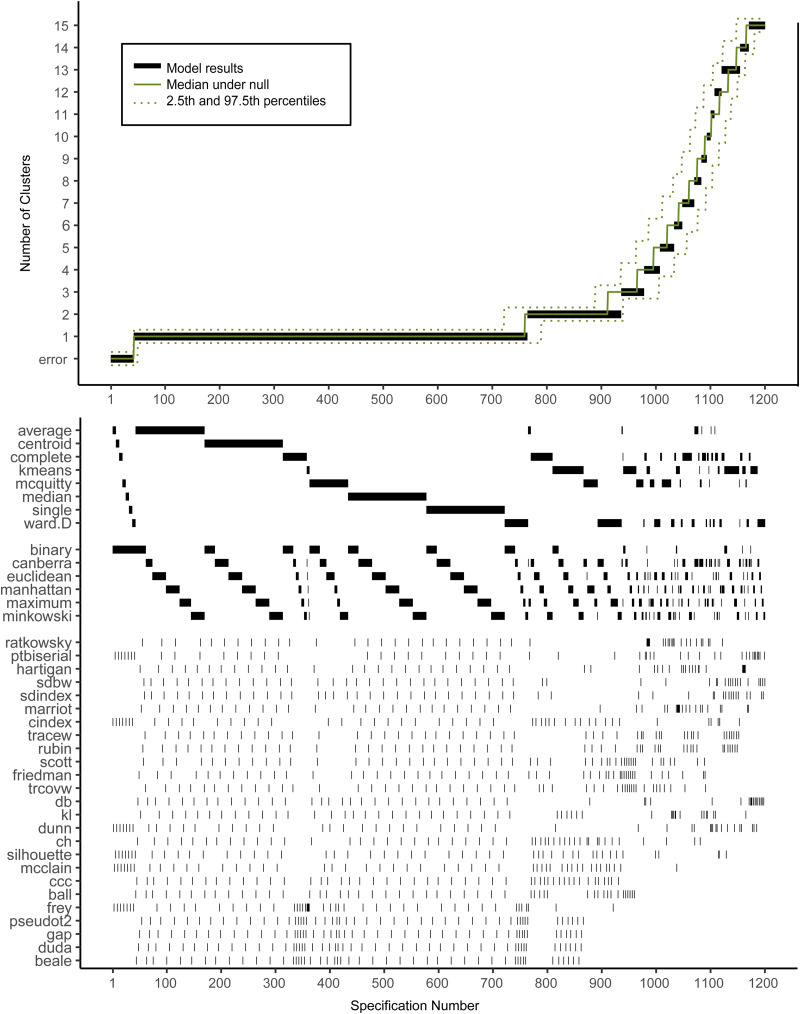
Descriptive Specification Curve in the sample with MDD subjects only, with small clusters (⩽1% of subjects) removed. *Each black dot in the top panel depicts an estimate of the optimal number of clusters (K) from a different specification; the dots vertically aligned in the lower panel indicate the analytic decisions behind those estimates. The green lines indicate the expected range of results at each position. N.B. this is not the expected range of the specific combination of options, but rather the range of the m^th^ smallest K.*

Figure 2 shows the descriptive specification curve for the MDD sample (online Supplementary Table S3 shows sample characteristics). Forty-two specifications resulted in an error (see online Supplementary Table S4). More than half of specifications (60.2%) resulted in models containing one or more small clusters (*n* ⩽ 1%) that were excluded (see online Supplementary Table S5). The resulting number of valid clusters was variable, although most models indicated no cluster structure (median = 1, IQR = 1–2). Interestingly, all analyses using the centroid, median or single-linkage algorithms indicated no clustering (*K* = 1), whereas single-cluster results were relatively uncommon for *k*-means, Ward and complete-linkage clustering algorithms (*m* = 5/150, *m* = 43/144 *m* = 44/144, respectively).

Based on Fig. 2, we cannot readily conclude that any cluster structure is present because the observed curve overlaps strongly with the curves based on the randomly drawn data. More specifically, although many specifications resulted in a solution with *K* ⩾ 2, this did not provide solid evidence for existing clusters as no result *K* was found more often in the real data compared to the random data.

Subject allocation showed limited stability, as indicated by different cluster sizes between model solutions and multiple distinct model solutions within each group of specifications with the same *K* (see Table 2). For example, for *K* = 2, the stability of subjects' cluster allocations between the most common two-cluster model (33.1%) and the second most common two-cluster solutions (11.6%) was only 56.8%.

**Table 2.** Stability measures of models with different numbers of clusters (*K*) for the MDD dataset

*K*	Number of models % of 1200, (*n*)	Distinct solutions,	Dominant solution^a^, % (*n*)	Unique solutions^b^, % (*n*)
1	60.2 (722)			
2	14.3 (172)	15	33.1 (57)	0.6 (1)
3	3.5 (42)	8	57.1 (24)	2.4 (1)
4	2.4 (29)	7	34.5 (10)	3.4 (1)
5	2.2 (26)	9	38.5 (10)	11.5 (3)
6	1.2 (15)	7	46.7 (7)	26.7 (4)
7	1.8 (22)	6	36.4 (8)	0 (0)
8	1.1 (13)	5	53.8 (7)	23.1 (3)
9	0.8 (10)	5	30 (3)	10 (1)
10	0.6 (7)	6	28.6 (2)	71.4 (5)
11	0.6 (7)	5	28.6 (2)	42.9 (3)
12	1.1 (13)	7	30.8 (4)	30.8 (4)
13	2.8 (34)	4	79.4 (27)	5.9 (2)
14	1.3 (16)	7	37.5 (6)	18.8 (3)
15	2.5 (30)	6	43.3 (13)	0 (0)
Error	3.5 (42)			

a The model solution (i.e. specific division of subjects) that occurs most often within the group of models containing *K* clusters.

b Number of model solutions that occur only once.

When healthy controls were included, the SCA was very similar (see online Supplementary Fig. S2 and Supplementary Tables S6 and S7). Although *K* = 2 was expected here, 2-cluster solutions were not found more often in this dataset compared to the random datasets.

### Simulated data

**Fig. 3.**
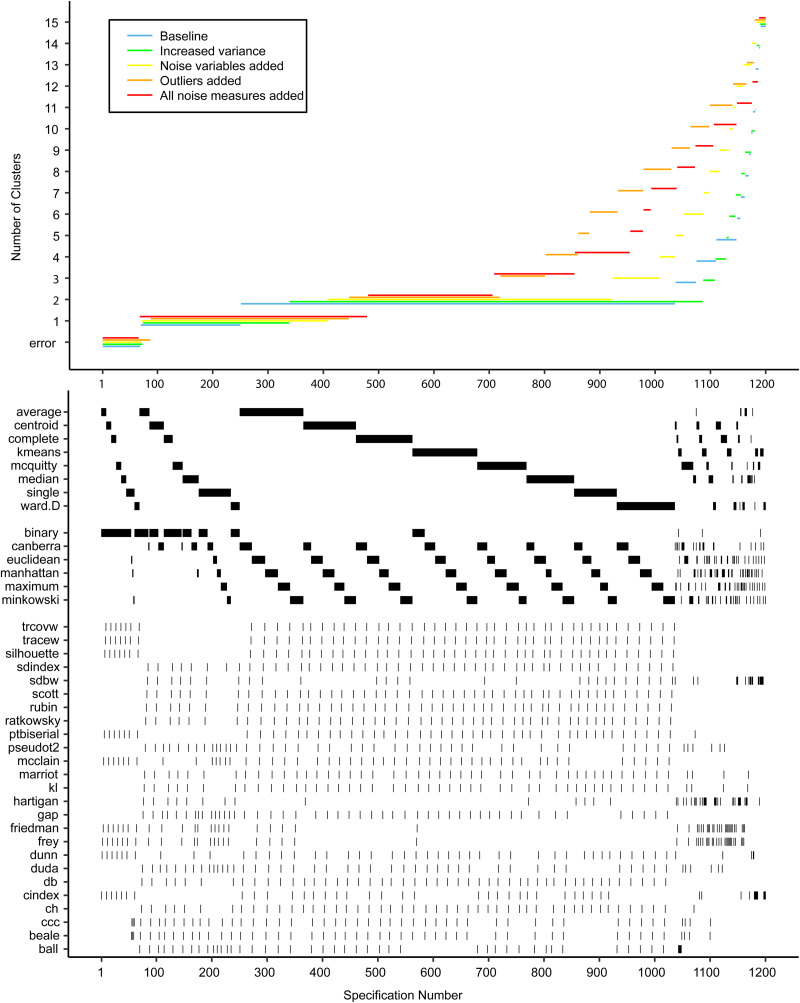
Specification curves based on simulated datasets with *K* = 2, with small clusters (⩽1% of subjects) removed. *Each dot in the top panel depicts an estimate of the optimal number of clusters (K) from a different specification; the dots vertically aligned in the lower panel indicate the analytic decisions behind the estimates of the baseline analysis. N.B. the analytic decisions behind the other analyses are not presented here.*

**Fig. 4.**
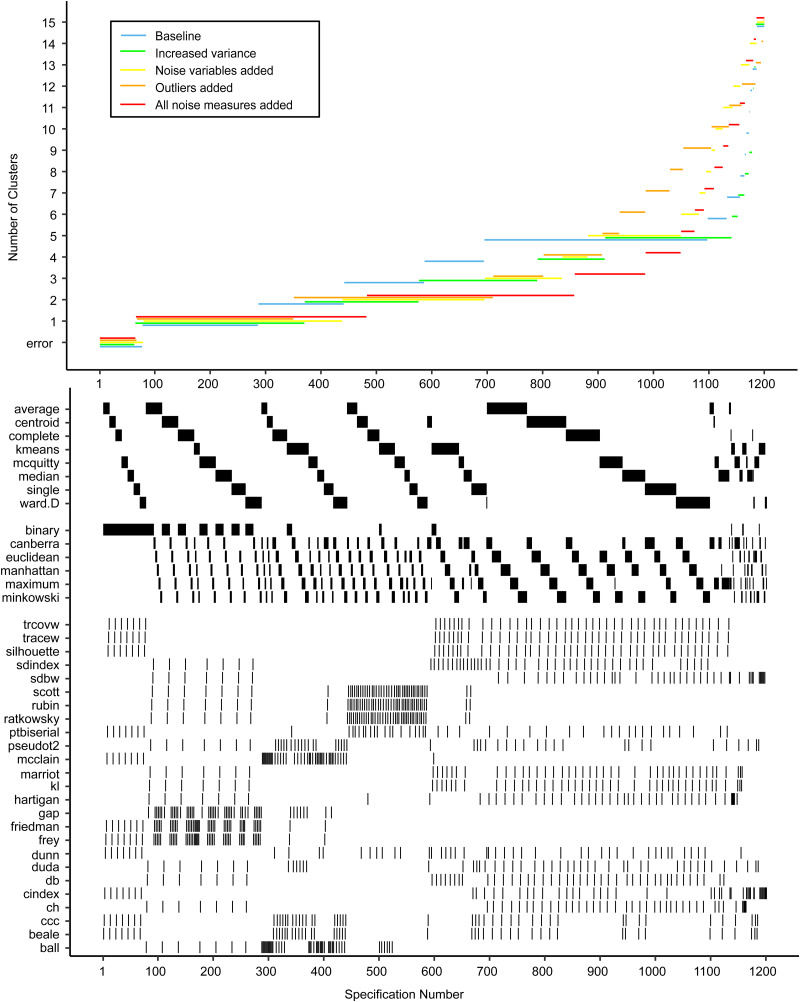
Specification curves based on simulated datasets with *K* = 5, with small clusters (⩽1% of subjects) removed. *Each dot in the top panel depicts an estimate of the optimal number of clusters (K) from a different specification; the dots vertically aligned in the lower panel indicate the analytic decisions behind the estimates of the baseline analysis. N.B. the analytic decisions behind the other analyses are not presented here.*

**Fig. 5.**
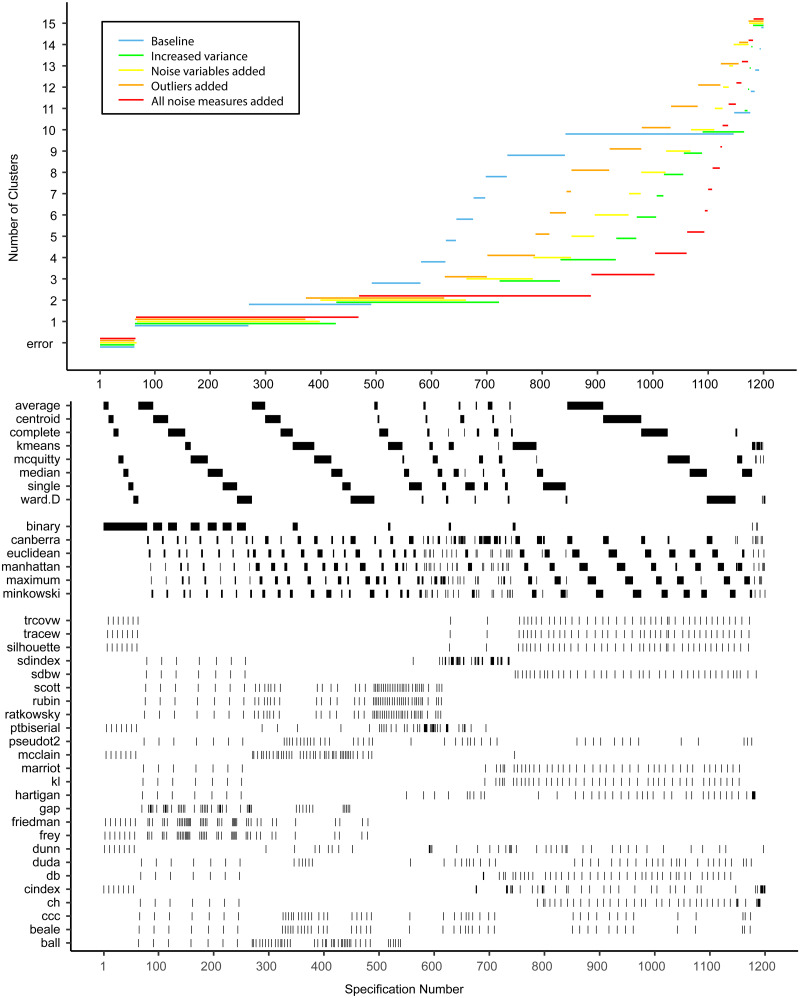
Specification curves based on simulated datasets with *K* = 10, with small clusters (⩽1% of subjects) removed. *Each dot in the top panel depicts an estimate of the optimal number of clusters (K) from a different specification; the dots vertically aligned in the lower panel indicate the analytic decisions behind the estimates of the baseline analysis. N.B. the analytic decisions behind the other analyses are not presented here.*

Figures 3–5 show the specification curves for simulated datasets. These showed that it is possible to detect the true number of clusters as the most consistent in the SCA, but that this is harder with a larger number of clusters. In the noise-free two-cluster data, most model specifications (65.5*%*) resulted in two clusters (see Fig. 2 and online Supplementary Tables S8 and S9). For the dataset with five and 10 clusters, these percentages were 33.6% (see Fig. 3 and online Supplementary Tables S10 and S11) and 25.4% (see Fig. 4 and online Supplementary Tables S12 and S13), respectively. Within specifications with the correct results, the classification accuracy was almost 100% for the three most common model solutions in each of the three noise-free datasets. Consequently, the stability of subject allocation was high between models.

Increasing the level of noise in the simulated datasets led to a decrease in correctly identified results in the SCA. SCAs in data with 20% noise variables showed a similar number of correct results as in datasets without noise (62.3, 33.6, and 25.4% respectively). However, transforming 20% of the sample to outliers did have a larger effect, especially in the two-and five-cluster datasets, where the number of correctly identified clusters in the SCA was similar to the SCA results obtained in datasets with added noise, outliers and cluster overlap (22.8% *v.* 19.1% and 2.6% *v.* 2.1%, respectively). Increasing the variance especially influenced the number of correctly identified results in the ten-cluster dataset: 3.6% correct results compared to 0.9% with added noise, outliers and increased variance.

## Discussion

We investigated the presence of data-driven biological clusters of depression and evaluated the effect of different model specifications on these findings. The cluster-analysis results based on our sample of MDD patients were very sensitive to the model specifications used. The SCA showed that the number of identified clusters was inconsistent, and that cluster allocation stability was low. Together, these observations indicated no robust cluster structure in the real dataset. This was also the case for the sample including healthy controls. Moreover, our analyses showed that many specifications will result in a cluster solution even when no structure is present in the data. The simulation study showed that it is possible for SCA to correctly identify clusters as the most consistent solution if they are present in the data, but that this becomes more difficult with large number of clusters and/or higher noise levels. Below, implications of these results are discussed.

As discussed in the introduction, the variability in results of previous cluster analyses raises inevitable questions about how much confidence we should put in results from a single cluster analysis, especially when this single analysis lacks replication in independent samples and clinical validation (e.g. differences in risk factors or course) (Beijers et al., 2019b; Marquand et al., 2016; Van Loo et al., 2012). Our study aimed to investigate if the faith in model results improves when SCA is applied. The simulation results are somewhat encouraging, but the lack of a robust cluster structure in the real dataset including the one with both MDD patients and healthy controls raises several concerns. How can we explain that the NESDA study found differences in biomarkers between cases and controls, but we do not find them in cluster analyses using the same biomarkers? Should the results bring into question the applicability of cluster techniques to biological data and therefore caution against any future use of such techniques?

It is possible that we did not find clusters in the real dataset because of technical issues. It could be, for instance, that the differences between cases and controls are too small to be picked up by cluster analysis, or that there is no sufficient correlation between the biomarkers or that the signal-to-noise ratio is insufficient for cluster detection.

Alternatively, the fact that the SCA was not able to distinguish between MDD patients and controls could indicate that the DSM categories cannot be validated using this specific type of biological data. Some, but not all, of the used biomarkers have been shown to be associated with depression before. For example, macrophage migration inhibitory factor, a pleiotropic cytokine, has been shown to be higher in MDD patients compared with controls in five out of six studies (Bloom & Al-Abed, 2014). Interleukin-1 receptor antagonist has also been shown to be increased in patients compared to controls (Maes et al., 1997; Milaneschi et al., 2009). The von Willebrand factor, a marker involved in hemostasis, was previously found to be increased in one study (Dominici et al., 2010), which is supported by earlier genetic findings of an association between depressive symptoms and a specific von Willebrand allele in cardiac patients (McCaffery et al., 2009). Pancreatic polypeptide, which was elevated in patients, has been linked to anorexia nervosa (Batterham et al., 2003), and another member of the pancreatic polypeptide family, peptide YY, was (marginally) positively related to depressive symptoms in older adults (Powell et al., 2014). The other individual markers that were identified by Bot et al. (2015) were not associated with MDD in previous studies, or have not previously been investigated. For instance, the lower levels of growth-regulated alpha protein were in contrast with a study that found higher levels – although this result was not significant in the validation cohort (Powell et al., 2014).

The simulation results indicated that it is difficult to identify stable/robust clusters, even when they do in fact exist, as they showed the analyses' sensitivity to data complexity (i.e. number of clusters), increased noise and/or the presence/number of outliers. This is also the case for analyses based on single specification simulations (Hands & Everitt, 1987). In some cases (i.e. low numbers of clusters, little noise), it is likely still possible to identify any robust clusters present with SCA. In that case, results should be considered much more reliable than that of a single analysis, because the former is robust to differences in model specifications. This has already been shown in social psychology, where for example the negative impact of racial bias on callback rates in job application processes has been shown to be robust, whereas increased death toll of female-named hurricanes was not (Simonsohn et al., 2020).

### Limitations

Our study should be considered in light of the following limitations. First, we used 31 biomarkers that were previously shown to differ between patients with current MDD and healthy controls using adjusted linear regression (Bot et al., 2015). It is possible that other biochemical markers are more suitable for finding clusters of MDD patients. Currently, it is unknown which measures are best suited for biological subtyping of depression (Beijers et al., 2019b), so it could also be that brain structure or functional connectivity (Hasler & Northoff, 2011) or genetic background (Flint & Kendler, 2014) could be more suitable for clustering MDD patients. Furthermore, it could be that inter-personal variations in psychiatric samples are better captured by continuous distributions [e.g. severity dimension(s)] rather than discrete clusters (Islam et al., 2018; van Loo et al., 2016; Wanders et al., 2016; Wardenaar, Wanders, ten Have, de Graaf, & de Jonge, 2017).

Second, SCA has traditionally been used in psychology to investigate the effects of using alternative regression models (Orben & Przybylski, 2019; Rohrer, Egloff, & Schmukle, 2017; Simonsohn et al., 2020). Cluster techniques are more complex. Two three-cluster solutions may be completely different in size and subject allocation, whereas a two- and a three-cluster solution may be partially overlapping. It is therefore important to keep in mind that this application of SCA focuses mainly on the resulting number of clusters and cluster stability, rather than the substantive interpretation of the clusters. Had we found an optimal number of clusters (*K*_optimal_) with a stable model solution, we would have investigated if the movement of subjects between models with *K*_optimal_ −1 and with *K*_optimal_ was stable. If this would have been the case, we would have investigated the movement of subjects between models with ever-decreasing *K*, in order to investigate if there was a stable division tree to be made all the way from *K* = 1 to *K*_optimal_.

Third, we used a limited number of model specifications for unsupervised learning. We focused on k-means clustering and hierarchical clustering because these are among the most commonly used methods across disorders (Marquand et al., 2016) and FMMs have been shown to have a number of issues that limit their usefulness for psychiatric classification. FMMs tend to detect groups with different severity levels, which is not always the aim of cluster analysis and local dependence between variables can obfuscate the results (Borsboom et al., 2016; Hagenaars, 1988; van Loo et al., 2016). Because there is insufficient evidence on which model clustering algorithms, distances and fit indices are most useful for a study like ours, we decided to study all of the potential model specifications and not to exclude any a priori. We decided to use the exhaustive list of options in the NbClust R-package, which was designed to gather all indices available in SAS and R packages together into a single one package as well as some newer indices that are not implemented anywhere else yet (Charrad et al., 2014).

Fourth, we did not perform a Monte Carlo SCA but rather used SCA to evaluate the result obtained in a single simulation study. There is no Monte Carlo element in our procedure as we did not seek to quantify clustering quality of SCA or a single specification *per se*. Rather, our simulations aimed to evaluate whether, in the presence of a known number of clusters in a population, SCA can robustly show this number across different model specifications. Therefore, we used simulated datasets to illustrate the use of SCA under different circumstances (different numbers of clusters, noise levels). In total, we only simulated 15 datasets (i.e. 2, 5 and 10 clusters with 5 different noise levels). We chose to simulate different noise levels by increasing the number of outliers (Saraçli et al., 2013), varying the number of informative variables (Clifford et al., 2011) and different degrees of separation between the clusters (Clifford et al., 2011; Ferreira & Hitchcock, 2009; Milligan, 1980) (i.e. increasing variance), but other methods of simulating noisy datasets also exist (Milligan, 1980).

Finally, it is important to remember that there are still many sources of variation left in our analyses, as can be seen in Fig. 1. For example, we limited our analysis to a single MDD dataset with a limited set of markers, because the primary focus was on the influence of model specifications on the results and not on the effects of different data-processing choices. Furthermore, we chose to exclude clusters smaller than 1% of the data, under the assumption that these are likely to represent methodological artifacts or outliers rather than true cluster structure in the data. Arguably, other approaches to such ‘nuisance clusters’ could have been equally valid. The same goes for the way we chose to estimate the model results under the null hypothesis for the real datasets.

## Conclusion

Clustering methods are important statistical techniques for psychiatric science to improve mental health care by identifying more homogeneous and biologically informed diagnostic categories. This study used SCA to investigate data-driven biological subtypes of MD and showed that the results of cluster analyses were heavily dependent on different model specifications. SCA can help to investigate the robustness of cluster analyses and identify stable clusters. As such, SCA is a useful technique that could aid the development of robust and replicable subtyping models in psychiatric disorders.

## Data

The dataset supporting the conclusions of this article is available in the Netherlands Study of Depression and Anxiety (NESDA) consortium at https://www.nesda.nl/nesda-english/. Due to privacy concerns, the data are available on reasonable request. The analysis code can be found on the Open Science Framework (https://osf.io/5jr28/).

## Supplementary material

For supplementary material accompanying this paper visit https://doi.org/10.1017/S0033291720002846. click here to view supplementary material
