# Investigating Data-driven Biological Subtypes of Psychiatric Disorders Using Specification-Curve Analysis – ERRATUM

**DOI:** 10.1017/S0033291721000660

**Published:** 2022-04

**Authors:** Lian Beijers, Hanna M. van Loo, Jan-Willem Romeijn, Femke Lamers, Robert A. Schoevers, Klaas J. Wardenaar

**Affiliations:** 1 Department of Psychiatry, University of Groningen, University Medical Center Groningen, Interdisciplinary Center Psychopathology and Emotion regulation (ICPE), Groningen, The Netherlands; 2 Faculty of Philosophy, University of Groningen, Groningen, The Netherlands; 3 GGZ inGeest and Department of Psychiatry, Amsterdam Public Health Research Institute, VU University Medical Center, Amsterdam, The Netherlands; 4 Department of Psychiatry, University of Groningen, University Medical Center Groningen, Research School of Behavioural and Cognitive Neurosciences, Groningen, The Netherlands

This article was published in Psychological Medicine with spelling mistake in its title. In the original the word ‘psychiatric’ was misspelled as ‘sychiatric’. This has now been updated.

The publisher apologises for this error.
