# Supplementary material for: Investigating data-driven biological subtypes of psychiatric disorders using specification-curve analysis
Source: Psychol Med. 2020 Aug 11;52(6):1089–100. doi: 10.1017/S0033291720002846 (PMC9069352; doi:10.1017/S0033291720002846)
Supplement: Supplementary file 1 [file S0033291720002846sup001.pdf]

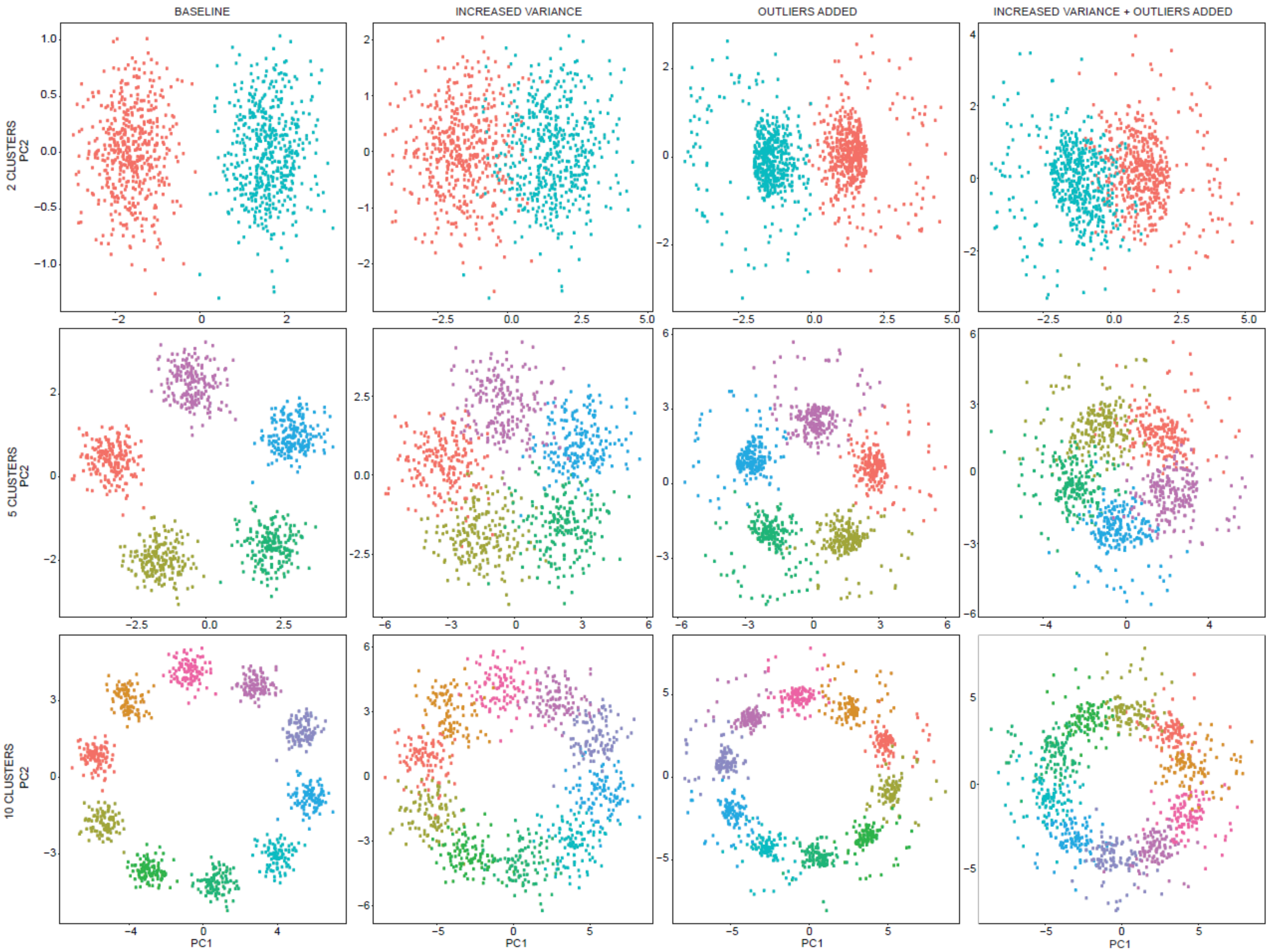

Supplementary Figure S1. The first two principal component coordinates of the simulated datasets with 2, 5 and 10 clusters. Plots are included for the datasets without noise, with added outliers, with increased variance and with the combination of added outliers and increased variance, as the noise variables were added at a later stage.

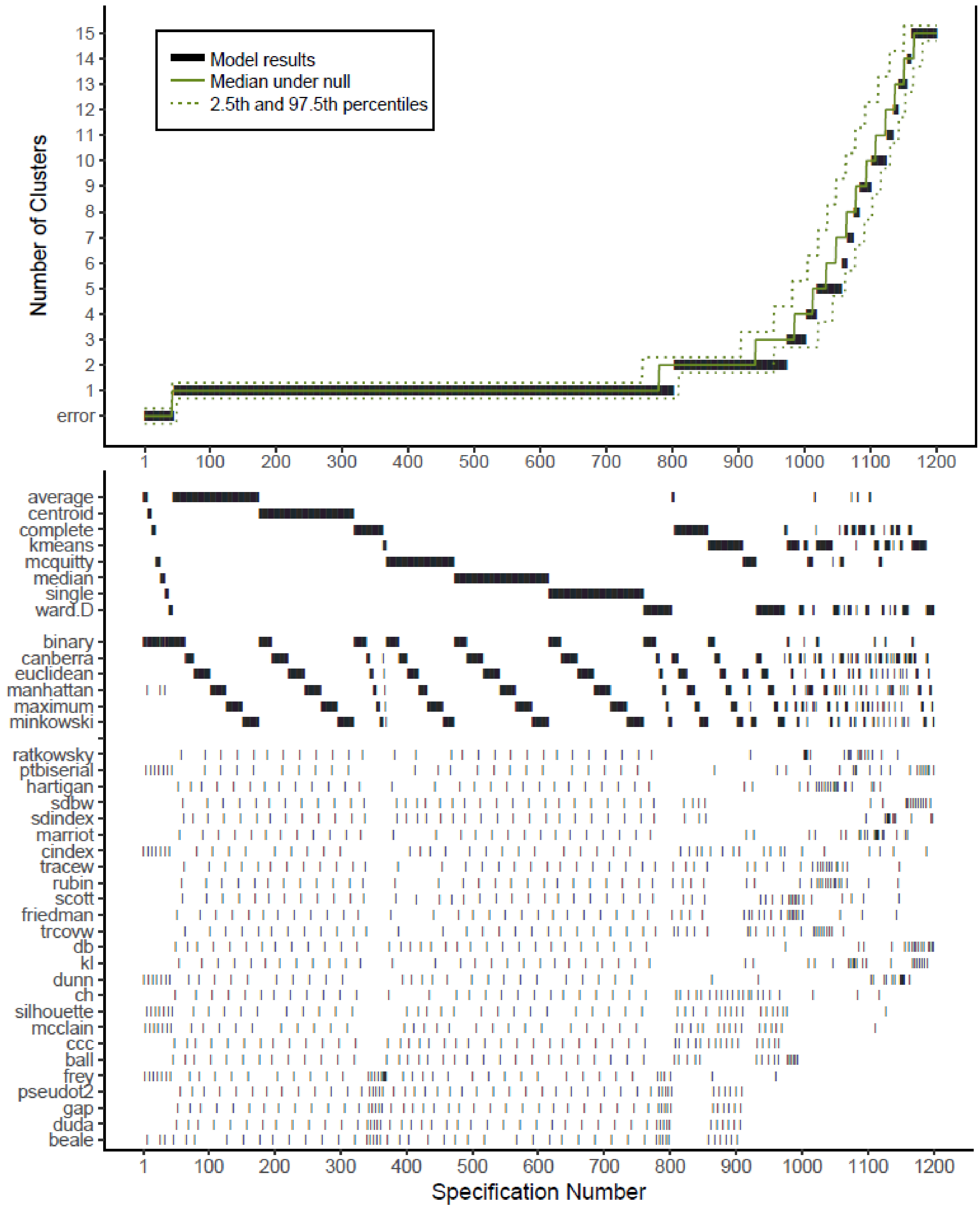

Supplementary F*igure S2. Descriptive Specification Curve in the sample with MDD subjects + healthy controls, with small clusters (≤1% of subjects) removed.* *Each black dot in the top panel depicts an estimate of the optimal number of clusters (K) from a different specification; the dots vertically aligned in the lower panel indicate the analytic decisions behind those estimates. The green lines indicate the expected range of results at each position. N.B. this is not the expected range of the specific combination of options, but rather the range of the* m^{th} *smallest K.*

Supplementary Table S1. Characteristics of biochemical analytes.

ANALYTE	*BIOLOGICAL PROCESS**	*SHORT DESCRIPTION*	*TOTAL MISSING*	*MISSING DUE TO DETECTION LIMIT*	*TRUE MISSING*
**ALPHA-1-ANTICHYMOTRYPSIN**	PM	Alpha-1-antichymotrypsin (AACT) is is an alpha globulin glycoprotein that is a member of the serpin superfamily. It inhibits the activity of certain proteases, such as cathepsin G that is found in neutrophils, and chymases found in mast cells. This acute phase protein is induced during inflammation, and produced in the liver.	0	0	0
**ALPHA-1-ANTITRYPSIN**	PM	Alpha-1-antitrypsin (AAT) is the most prominent protease belonging to the serpin superfamily. It is an acute phase protein produced in the liver. Associations of AAT with MDD have given mixed results (1, 2)	2	0	2
**CD40 ANTIGEN**	CC,ST	Cluster of differentiation 40 (CD40) is a costimulatory protein found on antigen presenting cells and is required for their activation.	2	0	2
**COMPLEMENT FACTOR H-RELATED PROTEIN 1**	IM	The complement system is important for host innate and adaptive immunity and mounts a protective immune response to invading microbes. Complement factor H-related protein 1 (CFHR1) is an inhibitor of the complement pathway that blocks C5 convertase activity and interferes with C5b surface deposition and membrane attack complex formation.(3)	0	0	0
**ENRAGE**	CC,ST	In view of its inflammatory function in innate immunity and its ability to detect a class of ligands through a common structural motif, RAGE (receptor for advanced glycation endproducts) is often referred to as a pattern recognition receptor. The interaction between RAGE and its ligands is thought to result in pro-inflammatory gene activation.(4) Due to an enhanced level of RAGE ligands in diabetes or other chronic disorders, this receptor is hypothesised to have a causative effect in a range of inflammatory diseases such as diabetic complications, Alzheimer's disease and even some tumors. ENRAGE (extracellular newly identified RAGE) is such a ligand.	0	0	0
**GROWTH-REGULATED ALPHA PROTEIN**	IM	Growth-regulated alpha protein (GROa) activates neutrophils.(5) It also plays a role in certain types of cancer, stimulating tumor-associated macrophages and cancer-associated fibroblasts.(6) Bot et al. (2015) observed lower levels of the GROa but another study found higher GROa levels in MDD patients in the discovery phase (not validated).(7)	0	0	0
**INTERLEUKIN-12P40**	IM	Subunit beta of interleukin 12 (also known as interleukin-12p40) is a common subunit of interleukin 12 and 13. Interleukin 12 is a cytokine that acts on T and natural killer cells, and has a broad array of biological activities. It is expressed by activated macrophages that serve as an essential inducer of Th1 cells development, and has been found to be important for sustaining a sufficient number of memory/effector Th1 cells to mediate long-term immunity.	62	61	1

**INTERLEUKIN-1 RECEPTOR ANTAGONIST**	CC,ST	Interleukin-1 recepter antagonist (IL-1RA) is an agent that binds non-productively to the cell surface interleukin-1 receptor, the same receptor that binds interleukin 1 (IL-1), preventing IL-1 from sending a signal to that cell. IL1Ra is secreted by various types of cells including immune cells, epithelial cells, and adipocytes, and is a natural inhibitor of the pro-inflammatory effect of IL1 beta.(8) Increased levels of interleukin-1 receptor antagonist have been found in patients with depression.(9)	29	28	1
**MACROPHAGE MIGRATION INHIBITORY FACTOR**	CC,ST	Macrophage migration inhibitory factor (MIF) is an important regulator of innate immunity. doi:10.1038/nri1200.Bacterial antigens stimulate white blood cells to release MIF into the blood stream. The circulating MIF binds to CD74 on other immune cells to trigger an acute immune response. Hence, MIF is classified as an inflammatory cytokine. Furthermore, glucocorticoids also stimulate white blood cells to release MIF and hence MIF partially counteracts the inhibitory effects that glucocorticoids have on the immune system. A systematic review showed that MIF was higher in persons with MDD or depressed mood, compared with non-depressed controls in five out of six studies.(10)	1	1	0
**LACTOYLGLUTATHIONE LYASE** (not included because of high correlation with MIF)	M	Lactoylglutathionase lyase (also known as glyoxalase I) catalyzes of the first step in the glyoxalase system, a critical two-step detoxification system for methylglyoxal. Methylglyoxal is produced naturally as a byproduct of normal biochemistry, but is highly toxic, due to its chemical reactions with proteins, nucleic acids, and other cellular components. Experiments suggest that methylglyoxal is preferentially toxic to proliferating cells, such as those in cancer.(11) Glyoxalase I is an attractive target for the development of drugs to treat infections by some parasitic protozoa, and cancer.(12)	2	2	0
**INSULIN GROWTH FACTOR-BINDING PROTEIN-5**	CC,ST	The six members of the insulin-like growth factor-binding protein family (IGFBP 1-6) are important components of the IGF (insulin-like growth factor) axis. In this capacity, they serve to regulate the activity of both IGF-I and -II polypeptide growth factors. IGFBP-5 also has an important role in controlling cell survival, differentiation and apoptosis.(13)	2	0	2
**UROKINASE-TYPE PLASMINOGEN ACTIVATOR RECEPTOR**	CC,ST	The urokinase receptor (also known as urokinase-type plasminogen activator receptor, uPAR), was originally identified as a saturable binding site for urokinase on the cell surface. Besides the primary ligand urokinase, uPAR interacts with several other proteins, among others: vitronectin, the uPAR associated protein (uPARAP) and the integrin family of membrane proteins. uPAR is a part of the plasminogen activation system, which in the healthy body is involved in tissue reorganization events such as mammary gland involution and wound healing. In order to be able to reorganize tissue, the old tissue must be able to be degraded. An important mechanism in this degradation is the proteolysis cascade initiated by the plasminogen activation system. uPAR binds urokinase and thus restricts plasminogen activation to the immediate vicinity of the cell membrane.(14)	10	9	1
**CATHEPSIN D**	PM	Cathepsin D is a lysosomal aspartyl protease.Over-expression of cathepsin D stimulates tumorigenicity and metastasis as well as initiation of tumor apoptosis. This protease has been regarded an independent marker of poor prognosis in breast cancer being correlated with the incidence of clinical metastasis.(15) It is also reported that there might be a strong effect for CTSD genotype on Alzheimer disease risk in males.(16)	3	0	3

**RECEPTOR TYROSINE-PROTEIN KINASE ERBB-3**	CC,ST	Receptor tyrosine-protein kinase erbB-3 is a member of the epidermal growth factor receptor (EGFR/ERBB) family of receptor tyrosine kinases. ERBB3 is expressed in normal adult human gastrointestinal tract, reproductive system, skin, nervous system, urinary tract, and endocrine system. Combined with ErbB2, it is implicated in growth, proliferation, chemotherapeutic resistance, and the promotion of invasion and metastasis.(17)	0	0	0
**HEPSIN**	PL	Hepsin is a type-II membrane-associated serine peptidase which plays a role in cell growth and development. The physiological substrate(s) of hepsin have not been defined.(18) Although the functional significance of this upregulation is unknown, overexpression of hepsin is frequently observed in human prostate cancer.(19)	0	0	0
**CELLULAR FIBRONECTIN**	CG	Fibronectin has numerous functions that ensure the normal functioning of vertebrate organisms. It is involved in cell adhesion, growth, migration, and differentiation. Cellular fibronectin is assembled into the extracellular matrix, an insoluble network that separates and supports the organs and tissues of an organism.	465	463	2
**MATRIX METALLOPROTEINASE-10**	PM	Matrix metalloproteinase-10 (MMP10, also known as stromelysin 2) is a calcium-dependent zinc-containing endopeptidase. MMPs are capable of degrading all kinds of extracellular matrix proteins, but also can process a number of bioactive molecules. Substrates for MMP10 include proteoglycans, laminin, fibronectin, elastin and collagen IV.	4	0	4
**MATRIX METALLOPROTEINASE-3**	PM	Matrix metalloproteinase-3 (MMP3, also known as stromelysin 1) is a calcium-dependent zinc-containing endopeptidase. MMPs are capable of degrading all kinds of extracellular matrix proteins, but also can process a number of bioactive molecules. Substrates for MMP3 include a number of CC chemokines. The MMP-3 enzyme also degrades collagen types II, III, IV, IX, and X, proteoglycans, fibronectin, laminin, and elastin. In addition, MMP-3 can also activate other MMPs such as MMP-1, MMP-7, and MMP-9, rendering MMP-3 crucial in connective tissue remodeling.(20) MMP-3 can weaken the blood-brain barrior and the blood-spinal cord barrier, which then allows for more neutrophils to infiltrate the brain and spinal cord at the site of inflammation.(21)	2	1	1
**TENASCIN C**	CC,ST	Tenascin C (TN-C) is expressed in the extracellular matrix of various tissues during development, disease or injury, and in restricted neurogenic areas of the central nervous system.(22) TN-C has been shown to be upregulated under pathological conditions caused by inflammation, infection, and tumorigenesis.(23) Tenascin-C has been shown to interact with fibronectin. A solid-state interaction between fibronectin and TN-C results in cellular upregulation of matrix metalloproteinase expression.(24)	1	0	1
**CARCINOEMBRYONIC ANTIGEN**	IM	Carcinoembryonic antigen (CEA) describes a set of highly related glycoproteins involved in cell adhesion. CEA is normally produced in gastrointestinal tissue during fetal development, but the production stops before birth. Consequently, CEA is usually present at very low levels in the blood of healthy adults (about 20 ng/mL). However, the serum levels are raised in some types of cancer, which means that it can be used as a tumor marker in clinical tests.(25) Serum levels can also be elevated in heavy smokers.	4	4	0

**ANGIOGENIN**	M	Angiogenin (ANG) is a small protein that stimulates the growth of new blood vessels through the process of angiogenesis. It is associated with cancer and neurological disease through angiogenesis and through activating gene expression that suppresses apoptosis.	3	0	3
**ANGIOPOIETIN 2**	CC,ST	Angiopoietin 2 is part of a family of vascular growth factors that play a role in embryonic and postnatal angiogenesis. Angiopoietin signaling most directly corresponds with angiogenesis, the process by which new arteries and veins form from preexisting blood vessels. Angiopoietin-2 promotes cell death and disrupts vascularization. Yet, when it is in conjunction with VEGF, it can promote neo-vascularization.	1	0	1
**VASCULAR ENDOTHELIAL GROWTH FACTOR**	CC,ST	Vascular endothelial growth factor (VEGF) is a signal protein produced by cells that stimulates the formation of blood vessels. VEGF is involved in both vasculogenesis (the de novo formation of the embryonic circulatory system) and angiogenesis (the growth of blood vessels from pre-existing vasculature). It is part of the system that restores the oxygen supply to tissues when blood circulation is inadequate such as in hypoxic conditions. Serum concentration of VEGF is high in bronchial asthma and diabetes mellitus.(26)	2	1	1
**APOLIPOPROTEIN A4**	T	Apolipoproteins are proteins that bind lipids (oil-soluble substances such as fat and cholesterol) to form lipoproteins. They transport the lipids through the lymphatic and circulatory systems. Apolipoproteins also serve as enzyme cofactors, receptor ligands, and lipid transfer carriers that regulate the metabolism of lipoproteins and their uptake in tissues. Intestinal fat absorption dramatically increases the synthesis and secretion of apo A-IV.	0	0	0
**APOLIPOPROTEIN D**	T	ApoD has also been shown to be an important link in the transient interaction between HDL and LDL particles and between HDL particles and cells. APOD is associated with neurological disorders and nerve injury, especially related to myelin sheath, and is elevated in patients with schizophrenia, bipolar disorder, and Alzheimer's disease.(27)	126	124	2
**FATTY ACID-BINDING PROTEIN, ADIPOCYTE**	CC,ST	The fatty-acid-binding proteins (FABPs) are a family of transport proteins for fatty acids and other lipophilic substances such as eicosanoids and retinoids.These proteins are thought to facilitate the transfer of fatty acids between extra- and intracellular membranes. It has been implicated in heart disease and diabetes(28) as well as asthma(29).	1	1	0
**PANCREATIC POLYPEPTIDE**	CC,ST	Pancreatic polypeptide (PPP) is a polypeptide secreted by PP cells in the endocrine pancreas predominantly in the head of the pancreas. The function of PP is to self-regulate pancreatic secretion activities (endocrine and exocrine); it also has effects on hepatic glycogen levels and gastrointestinal secretions. Its secretion in humans is increased after a protein meal, fasting, exercise, and acute hypoglycemia and is decreased by somatostatin and intravenous glucose. Plasma PP has been shown to be reduced in conditions associated with increased food intake and elevated in anorexia nervosa.(30)	1	0	1

**VON WILLEBRAND FACTOR**	PM	The von Willebrand factor (vWF) is a large multimeric glycoprotein present in blood plasma and produced constitutively as ultra-large vWF in endothelium (in the Weibel-Palade bodies), megakaryocytes (α-granules of platelets), and subendothelial connective tissue. It is involved in hemostasis. Increased plasma levels in a large number of cardiovascular, neoplastic, and connective tissue diseases are presumed to arise from adverse changes to the endothelium, and may contribute to an increased risk of thrombosis.(31) Increased levels of von Willebrand factor have been associated with MDD. (1)	20	18	2
**LUTEINIZING HORMONE** (not included because of high correlation with FSH)	CC,ST	Luteinizing hormone (LH) is a hormone produced by gonadotropic cells in the anterior pituitary gland. In females, an acute rise of LH ("LH surge") triggers ovulation and development of the corpus luteum. In males, where LH had also been called interstitial cell–stimulating hormone it stimulates Leydig cell production of testosterone. LH acts synergistically with FSH.	474	474	0
**FOLLICLE-STIMULATING HORMONE**	CC,ST	Follicle-stimulating hormone (FSH) is a gonadotropin, a glycoprotein polypeptide hormone. FSH is synthesized and secreted by the gonadotropic cells of the anterior pituitary gland, and regulates the development, growth, pubertal maturation, and reproductive processes of the body. FSH and LH work together in the reproductive system.	33	33	0
**CYSTATIN C**	PM	Cystatin C is mainly used as a biomarker of kidney function (which filter it from the blood). Cystatin C is found in virtually all tissues and body fluids and is a potent inhibitor of lysosomal proteinases and cysteine proteases. Recently, it has been studied for its role in predicting new-onset or deteriorating cardiovascular disease.(32) It also seems to play a role in brain disorders involving amyloid, such as Alzheimer's disease.(33)	0	0	0
**FETUIN-A**	CC,ST	Alpha-2-HS-glycoprotein (also know as Fetuin-A) belongs to the fetuin class of plasma binding proteins and is more abundant in fetal than adult blood. It is involved in several functions, such as endocytosis, brain development and the formation of bone tissue.(34) The protein is commonly present in the cortical plate of the immature cerebral cortex and bone marrow hemopoietic matrix, and it has therefore been postulated that it participates in the development of the tissues.	0	0	0
**PROSTASIN**	PM	Prostasin is a serine protease, found in the prostate gland, kidneys, bronchi, colon, liver, lungs, pancreas, and salivary glands. It is implicated in epithelial sodium channel regulation and may help regulate a variety of tissue functions that involve a sodium channel.(35) Higher levels of prostasin were previously observed in urine of MDD patients.(36)	1	0	1

Abbreviations: CC, cell-cell communication; CG, cell growth/maintentance; IM, immune response; M, metabolism; PL, proteolysis and peptidolysis; PM, protein metabolism ; ST, signal transduction;T, transport

** From the Human Protein Reference Database, according to Bot et al 2015.*

Supplmenterary Table S2. Included clustering methods, fit indices/inference tools and distance metrics.

**Clustering method**	**Fit index**	**Distance metric**
Hierarchical clustering	Ball*	Binary
Average	Beale	Canberra
Centroid	CCC	Euclidian
Complete	CH*	Manhattan
McQuitty	Cindex*	Maximum/Chebyshev
Median	DB*	Minowski
Centroid	Duda	
	Dunn*	
*K-means**	Frey	
	Friedman*	
	Gap	
	Hartigan*	
	KL*	
	Marriot*	
	Mcclain*	
	Pseudot2	
	Ptbiserial*	
	Ratkowsky*	
	Rubin*	
	Scott*	
	SDBW*	
	Sdindex*	
	Silhouette*	
	Tracew*	
	Trcovw*	

**methods or index incapable of testing against the null hypothesis of no clustering*

Supplementary Table S3. Baseline characteristics.

	**Current MDD (n = 688)**	**Healthy controls (n = 426)**	**P***
DEMOGRAPHICS AND COVARIATES			
Age, mean (sd)	41.87 (12.34)	38.95 (14.83)	0.007*
Sex, n female (%)	464 (67.4)	258 (60.6)	0.019*
BMI, mean (sd)	26 (5.64)	24.78 (4.63)	0.001*
Education, years (sd)	11.55 (3.18)	12.69 (3.14)	<0.001*
Number of chronic diseases			<0.001*
0, n (%)	268 (39.0)	262 (61.5)	
1, N (%)	237 (34.4)	115 (27.0)	
2, N (%)	124 (18.0)	33 (7.7)	
3+, N (%)	59 (8.6)	16 (3.8)	
LIFESTYLE FACTORS			
Physical Activity+ (IPAQ), median (IQR)	2.71 (4.13)	3.34 (4.03)	0.005*
smokers, N (%)	274 (39.8)	111 (26.1)	<0.001*
number of alcoholic drinks per week, median (IQR)	2.42 (8.10)	3.74 (7.69)	<0.001*
Depression severity (IDS), mean (sd)	31.53 (12.61)	8.25 (7.25)	<0.001*
Anxiety severity (BAI), mean (sd)	17.06 (10.94)	3.93 (4.74)	<0.001*
INTERNALIZING COMORBIDITY, n (%)			
Dysthymia	147 (21.4)	N.A.	NA
Gad	190 (27.6)	N.A.	NA
Social phobia	229 (33.3)	N.A.	NA
Panic disorder w. agoraphobia	142 (20.6)	N.A.	NA
Panic disorder w.o. agoraphobia	94 (13.7)	N.A.	NA
Agoraphobia	20 (7.3)	N.A.	NA
MEDICATION			
SSRI, N (%)	311 (30.7)	3 (0.7)	NA
TCA, N (%)	29 (4.2)	0 (0)	NA
Other AD, N (%)	77 (11.2)	0 (0)	NA
DDD SSRI, mean (sd)	1.47 (0.9)	1 (0)	NA
DDD TCA, mean (sd)	1.10 (0.6)	0 (0)	NA
DDD Other ad, mean (sd)	1.36 (0.8)	0 (0)	NA

AD, anti-depressive medication; AUDIT, alcohol use disorder identification test; BAI, Beck Anxiety Inventory; BMI, body mass index; GAD, generalized anxiety disorder; IDS, Inventory of Depressive Symptomatology; IPAQ, International Physical Activity Questionnaire; IQR, inter quartile range; MDD, major depression, NA, not applicable; SSRI, selective serotonin reuptake inhibitors; TCA, tricyclic antidepressants.

+ expressed in 1000 metabolic equivalent minutes per week

**Significant differences by class (at α < 0.05 corrected using the False Discovery Rate controlling method), based on ANOVA for continuous variables, Kruskal-Wallis tests for non-normally distributed variables and chi-square test for categorical variables.*

Supplementary Table S4. Overview of errors.

method	distance	index	Error occurred in datasets:					Error[1]
			2 cluster simulation (without noise)	5 cluster simulation (without noise)	10 cluster simulation (without noise)	Depression	Depression + HC	
average	binary	beale		x				1
average	binary	ccc		x				1
average	binary	cindex	x	x	x	x	x	2
average	binary	dunn	x	x	x	x	x	2
average	binary	frey	x	x	x	x	x	3
average	binary	friedman	x	x	x			1
average	binary	mcclain	x	x	x	x	x	2
average	binary	ptbiserial	x	x	x	x	x	2
average	binary	silhouette	x	x	x	x	x	2
average	binary	tracew	x	x	x			1
average	binary	trcovw	x	x	x			1
average	manhattan	beale					x	4
centroid	binary	beale		x				1
centroid	binary	ccc		x				1
centroid	binary	cindex	x	x	x	x	x	2
centroid	binary	dunn	x	x	x	x	x	2
centroid	binary	frey	x	x	x	x	x	3
centroid	binary	friedman	x	x	x			1
centroid	binary	mcclain	x	x	x	x	x	2
centroid	binary	ptbiserial	x	x	x	x	x	2
centroid	binary	silhouette	x	x	x	x	x	2
centroid	binary	tracew	x	x	x			1
centroid	binary	trcovw	x	x	x			1
complete	binary	beale		x				1
complete	binary	ccc		x				1
complete	binary	cindex	x	x	x	x	x	2
complete	binary	dunn	x	x	x	x	x	2
complete	binary	frey	x	x	x	x	x	3
complete	binary	friedman	x	x	x			1
complete	binary	mcclain	x	x	x	x	x	2
complete	binary	ptbiserial	x	x	x	x	x	2
complete	binary	silhouette	x	x	x	x	x	2
complete	binary	tracew	x	x	x			1
complete	binary	trcovw	x	x	x			1
mcquitty	binary	beale		x				1
mcquitty	binary	ccc		x				1
mcquitty	binary	cindex	x	x	x	x	x	2
mcquitty	binary	dunn	x	x	x	x	x	2
mcquitty	binary	frey	x	x	x	x	x	3
mcquitty	binary	friedman	x	x	x			1
mcquitty	binary	mcclain	x	x	x	x	x	2

mcquitty	binary	ptbiserial	x	x	x	x	x	2
mcquitty	binary	silhouette	x	x	x	x	x	2
mcquitty	binary	tracew	x	x	x			1
mcquitty	binary	trcovw	x	x	x			1
mcquitty	manhattan	beale					x	1
median	binary	beale		x				1
median	binary	ccc		x				1
median	binary	cindex	x	x	x	x	x	2
median	binary	dunn	x	x	x	x	x	2
median	binary	frey	x	x	x	x	x	3
median	binary	friedman	x	x	x			1
median	binary	mcclain	x	x	x	x	x	2
median	binary	ptbiserial	x	x	x	x	x	2
median	binary	silhouette	x	x	x	x	x	2
median	manhattan	beale					x	1
median	binary	tracew	x	x	x			1
median	binary	trcovw	x	x	x			1
single	binary	beale		x				1
single	binary	ccc		x				1
single	binary	cindex	x	x	x	x	x	2
single	binary	dunn	x	x	x	x	x	2
single	binary	frey	x	x	x	x	x	3
single	binary	friedman	x	x	x			1
single	binary	mcclain	x	x	x	x	x	2
single	binary	ptbiserial	x	x	x	x	x	2
single	binary	silhouette	x	x	x	x	x	2
single	binary	tracew	x	x	x			1
single	binary	trcovw	x	x	x			1
single	euclidean	beale	x					1
single	euclidean	ccc	x					2
single	manhattan	beale	x					1
single	manhattan	ccc	x					1
single	minkowski	beale	x					1
single	minkowski	ccc	x					1
ward.D	binary	beale		x				1
ward.D	binary	ccc		x				1
ward.D	binary	cindex	x	x	x	x	x	2
ward.D	binary	dunn	x	x	x	x	x	2
ward.D	binary	frey	x	x	x	x	x	3
ward.D	binary	friedman	x	x	x			1
ward.D	binary	mcclain	x	x	x	x	x	2
ward.D	binary	ptbiserial	x	x	x	x	x	2
ward.D	binary	silhouette	x	x	x	x	x	2
ward.D	binary	tracew	x	x	x			1
ward.D	binary	trcovw	x	x	x			1

[1]*Error 1: "The TSS matrix is indefinite. There must be too many missing values. The index cannot be calculated." This error appears when the input data has negative Eigenvalues.*

Error 2: NaNs were produced when calculating the values for these indices. This lead to a 0 as the best value for the index, combined the best number of classes lowest or highest according to the decision rule for each index.
Error 3: Error in if (res[ncF - min_nc + 1, 21] < 1) { : missing value where TRUE/FALSE needed" This error appears when the Frey index has not been successfully calculated, which could happen for several reasons.
Error 4: "Error in cutree(hc, k = best.nc) : object 'best.nc' not found" This error appears when there is no decision on the best number of clusters for some reason. This usually means that no model met the required threshold (e.g. the decision rule for the Beale index is the smallest number of clusters where the value of the index >= 0.1)

Supplementary Table S5. Comparing model results with and without removal of small (≤1%) clusters in the MDD dataset.

	1	*2*	*3*	*4*	*5*	*6*	*7*	*8*	*9*	*10*	*11*	*12*	*13*	*14*	*15*
1	**191**														
2	241	**157**													
3	26	6	**27**												
4	6	1	0	**17**											
5	5	2	2	4	**9**										
6	24	0	3	0	0	**11**									
7	19	0	1	0	0	2	**12**								
8	18	2	0	0	0	0	8	**2**							
9	18	0	0	0	2	0	2	4	**7**						
10	24	0	0	2	0	0	0	7	2	**3**					
11	9	0	6	0	2	0	0	0	0	3	**4**				
12	8	0	0	0	0	0	0	0	1	1	0	**8**			
13	52	0	3	0	1	0	0	0	0	0	0	0	**33**		
14	13	0	0	6	0	1	0	0	0	0	1	0	1	**11**	
15	68	4	0	0	12	1	0	0	0	0	2	5	0	5	**30**

The rows show how the model results with each number of clusters (K) are distributed after removal of small (≤1%) clusters. The columns show how many models had different numbers of clusters before removal of small (≤1%) clusters for each number of clusters (K) used in the SCA plots. The diagonal shows the number of models that only contained large (>1% clusters).

Supplementary Table S6. Comparing model results with and without removal of small (≤1%) clusters in the combined MDD patient + healthy control dataset.

	1	***2***	***3***	***4***	***5***	***6***	***7***	***8***	***9***	***10***	***11***	***12***	***13***	***14***	***15***
1	**181**														
2	278	**147**													
3	34	3	**27**												
4	17	8	2	**8**											
5	12	0	0	0	**34**										
6	4	4	0	0	1	**1**									
7	18	4	0	0	0	1	**5**								
8	10	0	0	0	0	0	5	**3**							
9	34	0	0	0	2	0	0	1	**5**						
10	10	0	0	0	1	6	0	2	2	**10**					
11	6	0	0	0	0	0	0	0	4	0	**9**				
12	36	0	0	5	0	0	0	0	2	0	0	**5**			
13	13	0	0	0	0	0	0	3	1	0	0	0	**13**		
14	22	4	0	1	0	0	0	0	4	3	0	0	0	**6**	
15	82	1	0	2	0	0	0	0	0	10	1	3	0	0	**39**

The rows show how the model results with each number of clusters (K) are distributed after removal of small (≤1%) clusters. The columns show how many models had different numbers of clusters before removal of small (≤1%) clusters for each number of clusters (K) used in the SCA plots. The diagonal shows the number of models that only contained large (>1% clusters).

Supplementary Table S7. Stability measures of models with different numbers of clusters (K) in the combined MDD patient + healthy control dataset.

K	Number of models % of 1200, (n)	Distinct solutions,	Dominant solution*, % (n)	Unique solutions+, % (n)
**1**	63.1 (757)			
**2**	14.2 (171)	13	31 (53)	0.6 (1)
**3**	2.4 (29)	5	62.1 (18)	3.4 (1)
**4**	1.3 (16)	4	37.5 (6)	0 (0)
**5**	3.2 (38)	7	65.8 (25)	5.3 (2)
**6**	0.7 (8)	3	75 (6)	25 (2)
**7**	0.8 (10)	4	40 (4)	20 (2)
**8**	0.8 (9)	7	22.2 (2)	55.6 (5)
**9**	1.5 (18)	9	22.2 (4)	16.7 (3)
**10**	1.9 (23)	8	30.4 (7)	8.7 (2)
**11**	0.8 (10)	3	80 (8)	20 (2)
**12**	0.7 (8)	5	37.5 (3)	37.5 (3)
**13**	1.1 (13)	4	46.2 (6)	7.7 (1)
**14**	0.5 (6)	3	50 (3)	16.7 (1)
**15**	3.2 (39)	6	56.4 (22)	0 (0)
**Error**	3.8 (45)			

**the model solution (i.e. specific division of subjects) that occurs most often within the group of models containing K clusters*

+number of model solutions that occur only once

Supplementary Table S8. Comparing model results with and without removal of small (≤1%) clusters in the dataset with 2 simulated clusters (without noise/outliers).

	1	*2*	*3*	*4*	*5*	*6*	*7*	*8*	*9*	*10*	*11*	*12*	*13*	*14*	*15*
1	**91**														
2	45	**651**													
3	1	64	**28**												
4	1	42	1	**21**											
5	0	15	5	5	**23**										
6	7	2	0	4	3	**3**									
7	7	1	1	4	2	1	**4**								
8	0	5	1	0	1	0	0	**0**							
9	7	1	0	0	2	3	3	1	**0**						
10	7	0	0	0	2	0	1	3	0	**0**					
11	0	0	0	0	2	0	0	3	0	0	**1**				
12	0	0	0	1	0	0	0	0	0	0	2	**0**			
13	0	0	1	1	0	2	0	0	2	0	0	0	**5**		
14	0	0	0	0	0	0	0	0	0	1	1	1	0	**0**	
15	28	4	1	0	0	0	0	1	2	0	2	0	1	3	**10**

The rows show how the model results with each number of clusters (K) are distributed after removal of small (≤1%) clusters. The columns show how many models had different numbers of clusters before removal of small (≤1%) clusters for each number of clusters (K) used in the SCA plots. The diagonal shows the number of models that only contained large (>1% clusters).

Supplementary Table S9. Stability measures of models with different numbers of clusters (K) in the dataset with 2 simulated clusters (without noise/outliers).

K	Number of models % of 1200, (n)	Distinct solutions,	Dominant solution*, % (n)	Unique solutions+, % (n)
**1**	15.1 (181)			
**2**	65.5 (786)	12	33.8 (266)	0 (0)
**3**	3.2 (38)	12	36.8 (14)	15.8 (6)
**4**	3 (36)	13	22.2 (8)	11.1 (4)
**5**	3.1 (37)	12	21.6 (8)	2.7 (1)
**6**	0.6 (7)	5	28.6 (2)	42.9 (3)
**7**	0.7 (8)	5	25 (2)	25 (2)
**8**	0.6 (7)	5	28.6 (2)	42.9 (3)
**9**	0.3 (4)	3	50 (2)	50 (2)
**10**	0.2 (3)	3	33.3 (1)	100 (3)
**11**	0.4 (5)	4	40 (2)	60 (3)
**12**	0 (0)			
**13**	0.5 (6)	2	83.3 (5)	16.7 (1)
**14**	0.2 (3)	2	66.7 (2)	33.3 (1)
**15**	0.8 (10)	4	60 (6)	20 (2)
**Error**	5.8 (69)			

**the model solution (i.e. specific division of subjects) that occurs most often within the group of models containing K clusters*

+number of model solutions that occur only once

Supplementary Table S10. Comparing model results with and without removal of small (≤1%) clusters in dataset with 5 simulated clusters (without noise/outliers).

	1	*2*	*3*	*4*	*5*	*6*	*7*	*8*	*9*	*10*	*11*	*12*	*13*	*14*	*15*
1	**146**														
2	42	**155**													
3	0	0	**145**												
4	0	0	0	**106**											
5	0	0	0	2	**325**										
6	0	0	0	0	56	**24**									
7	0	0	0	0	9	1	**15**								
8	0	0	0	0	6	1	5	**7**							
9	0	0	0	0	0	4	1	0	**1**						
10	7	0	0	0	1	0	0	0	0	**0**					
11	0	0	0	0	0	0	0	1	0	0	**0**				
12	0	0	0	0	0	0	0	2	0	2	0	**2**			
13	21	0	0	0	2	0	1	0	0	1	0	0	**8**		
14	0	0	0	0	0	1	0	0	0	2	0	0	0	**0**	
15	14	0	0	0	3	5	0	0	0	2	0	3	1	0	**14**

The rows show how the model results with each number of clusters (K) are distributed after removal of small (≤1%) clusters. The columns show how many models had different numbers of clusters before removal of small (≤1%) clusters for each number of clusters (K) used in the SCA plots. The diagonal shows the number of models that only contained large (>1% clusters).

Supplementary Table S11. Stability measures of models with different numbers of clusters (K) in in the dataset with 5 simulated clusters (without noise/outliers).

K	Number of models % of 1200, (n)	Distinct solutions,	Dominant solution*, % (n)	Unique solutions+, % (n)
**1**	17.4 (209)			
**2**	12.9 (155)	10	34.8 (54)	0 (0)
**3**	12.1 (145)	9	39.3 (57)	0 (0)
**4**	9 (108)	7	45.4 (49)	0.9 (1)
**5**	33.6 (403)	18	29.8 (120)	1 (4)
**6**	2.9 (35)	11	37.1 (13)	14.3 (5)
**7**	2 (24)	8	33.3 (8)	16.7 (4)
**8**	0.7 (8)	2	87.5 (7)	12.5 (1)
**9**	0.2 (3)	2	66.7 (2)	33.3 (1)
**10**	0.4 (5)	3	40 (2)	20 (1)
**11**	0.2 (2)	1	100 (2)	0 (0)
**12**	0.3 (4)	3	50 (2)	50 (2)
**13**	0.7 (8)	1	100 (8)	0 (0)
**14**	0 (0)			
**15**	1.2 (14)	4	78.6 (11)	21.4 (3)
**Error**	6.4 (77)			

**the model solution (i.e. specific division of subjects) that occurs most often within the group of models containing K clusters*
+number of model solutions that occur only once

Supplementary Table S12. Comparing model results with and without removal of small (≤1%) clusters in the dataset with 10 simulated clusters (without noise/outliers).

	1	***2***	***3***	***4***	***5***	***6***	***7***	***8***	***9***	***10***	***11***	***12***	***13***	***14***	***15***
1	**129**														
2	42	**222**													
3	0	0	**88**												
4	0	0	1	**45**											
5	0	0	0	0	**15**										
6	0	0	0	0	4	**30**									
7	0	0	0	0	0	0	**14**								
8	0	0	0	0	0	1	0	**29**							
9	0	0	0	0	0	0	6	7	**54**						
10	7	0	0	0	0	0	2	1	17	**237**					
11	0	0	0	0	0	0	0	2	17	29	**15**				
12	0	0	0	0	0	0	0	0	0	24	2	**18**			
13	28	0	0	0	0	0	0	0	8	5	4	2	**7**		
14	0	0	0	0	0	0	0	0	2	2	0	0	0	**3**	
15	7	0	0	0	0	0	0	0	7	2	0	3	1	0	**5**

The rows show how the model results with each number of clusters (K) are distributed after removal of small (≤1%) clusters. The columns show how many models had different numbers of clusters before removal of small (≤1%) clusters for each number of clusters (K) used in the SCA plots. The diagonal shows the number of models that only contained large (>1% clusters).

Supplementary Table S13. Stability measures of models with different numbers of clusters (K) in the dataset with 10 simulated clusters (without noise/outliers).

K	Number of models % of 1200, (n)	Distinct solutions,	Dominant solution*, % (n)	Unique solutions+, % (n)
**1**	17.2 (206)			
**2**	18.5 (222)	11	38.7 (86)	0 (0)
**3**	7.4 (89)	11	29.2 (26)	1.1 (1)
**4**	3.8 (45)	14	15.6 (7)	13.3 (6)
**5**	1.6 (19)	6	47.4 (9)	10.5 (2)
**6**	2.6 (31)	8	32.3 (10)	12.9 (4)
**7**	1.8 (22)	9	27.3 (6)	18.2 (4)
**8**	3.2 (39)	15	17.9 (7)	12.8 (5)
**9**	8.8 (105)	16	41 (43)	3.8 (4)
**10**	25.4 (305)	35	13.8 (42)	2 (6)
**11**	2.5 (30)	8	26.7 (8)	10 (3)
**12**	0.7 (8)	2	75 (6)	0 (0)
**13**	0.7 (8)	2	87.5 (7)	12.5 (1)
**14**	0.2 (3)	2	66.7 (2)	33.3 (1)
**15**	0.4 (5)	4	40 (2)	60 (3)
**Error**	5.2 (63)			

**the model solution (i.e. specific division of subjects) that occurs most often within the group of models containing K clusters*

+number of model solutions that occur only once

References

1. Domenici E, Willé DR, Tozzi F, Prokopenko I, Miller S, McKeown A, *et al.* (2010): Plasma protein biomarkers for depression and schizophrenia by multi analyte profiling of case-control collections. *PLoS One*. . doi: 10.1371/journal.pone.0009166.

2. Papakostas GI, Shelton RC, Kinrys G, Henry ME, Bakow BR, Lipkin SH, *et al.* (2013): Assessment of a multi-assay, serum-based biological diagnostic test for major depressive disorder: a pilot and replication study. *Mol Psychiatry*. 18: 332–339.

3. Heinen S, Hartmann A, Lauer N, Wiehl U, Dahse HM, Schirmer S, *et al.* (2009): Factor H-related protein 1 (CFHR-1) inhibits complement C5 convertase activity and terminal complex formation. *Blood*. . doi: 10.1182/blood-2009-02-205641.

4. Bierhaus A, Schiekofer S, Schwaninger M, Andrassy M, Humpert PM, Chen J, *et al.* (2001): Diabetes-associated sustained activation of the transcription factor nuclear factor-κB. *Diabetes*. . doi: 10.2337/diabetes.50.12.2792.

5. Cai S, Batra S, Lira SA, Kolls JK, Jeyaseelan S (2010): CXCL1 Regulates Pulmonary Host Defense to Klebsiella Infection via CXCL2, CXCL5, NF- B, and MAPKs. *J Immunol*. . doi: 10.4049/jimmunol.0903843.

6. Miyake M, Hori S, Morizawa Y, Tatsumi Y, Nakai Y, Anai S, *et al.* (2016): CXCL1-Mediated Interaction of Cancer Cells with Tumor-Associated Macrophages and Cancer-Associated Fibroblasts Promotes Tumor Progression in Human Bladder Cancer. *Neoplasia (United States)*. . doi: 10.1016/j.neo.2016.08.002.

7. Gottschalk MG, Cooper JD, Chan MK, Bot M, Penninx B, Bahn S (2015): Discovery of serum biomarkers predicting development of a subsequent depressive episode in social anxiety disorder. *Brain Behav Immun*. 48: 123–131.

8. Perrier S, Darakhshan F, Hajduch E (2006): IL-1 receptor antagonist in metabolic diseases: Dr Jekyll or Mr Hyde? *FEBS Lett*. . doi: 10.1016/j.febslet.2006.10.061.

9. Maes M, Bosmans E, De Jongh R, Kenis G, Vandoolaeghe E, Neels H (1997): Increased serum IL-6 and IL-1 receptor antagonist concentrations in major depression and treatment resistant depression. *Cytokine*. . doi: 10.1006/cyto.1997.0238.

10. Bloom J, Al-Abed Y (2014): MIF: Mood Improving/Inhibiting Factor? *J Neuroinflammation*. . doi: 10.1186/1742-2094-11-11.

11. Ayoub FM, Allen RE, Thornalley PJ (1993): Inhibition of proliferation of human leukaemia 60 cells by methylglyoxal in vitro. *Leuk Res*. . doi: 10.1016/0145-2126(93)90094-2.

12. Thornalley PJ (1993): The glyoxalase system in health and disease. *Mol Aspects Med*. . doi: 10.1016/0098-2997(93)90002-U.

13. Beattie J, Allan GJ, Lochrie JD, Flint DJ (2006): Insulin-like growth factor-binding protein-5 (IGFBP-5): a critical member of the IGF axis. *Biochem J*. . doi: 10.1042/bj20060086.

14. Ploug M (2005): Structure-Function Relationships in the Interaction Between the Urokinase- Type Plasminogen Activator and Its Receptor. *Curr Pharm Des*. . doi: 10.2174/1381612033454630.

15. Garcia M, Platet N, Liaudet E, Laurent V, Derocq D, Brouillet JP, Rochefort H (1996): Biological and clinical significance of cathepsin D in breast cancer metastasis. *Stem Cells*. . doi: 10.1002/stem.140642.

16. Menzer G, Müller-Thomsen T, Meins W, Alberici A, Binetti G, Hock C, *et al.* (2001): Non-replication

of association between cathepsin D genotype and late onset alzheimer disease. *Am J Med Genet - Neuropsychiatr Genet*. . doi: 10.1002/ajmg.1204.

17. Holbro T, Beerli RR, Maurer F, Koziczak M, Barbas CF, Hynes NE (2003): The ErbB2/ErbB3 heterodimer functions as an oncogenic unit: ErbB2 requires ErbB3 to drive breast tumor cell proliferation. *Proc Natl Acad Sci*. . doi: 10.1073/pnas.1537685100.

18. Wu Q, Peng J (2013): Chapter 652 - Hepsin. *Handb Proteolytic Enzym*. . doi: http://dx.doi.org/10.1016/B978-0-12-382219-2.00652-9.

19. Irshad S, Abate-Shen C (2013): Modeling prostate cancer in mice: Something old, something new, something premalignant, something metastatic. *Cancer Metastasis Rev*. . doi: 10.1007/s10555-012-9409-1.

20. Ye S, Eriksson P, Hamsten A, Kurkinen M, Humphries SE, Henney AM (1996): Progression of coronary atherosclerosis is associated with a common genetic variant of the human stromelysin-1 promoter which results in reduced gene expression. *J Biol Chem*. . doi: 10.1074/jbc.271.22.13055.

21. Gurney KJ, Estrada EY, Rosenberg GA (2006): Blood-brain barrier disruption by stromelysin-1 facilitates neutrophil infiltration in neuroinflammation. *Neurobiol Dis*. . doi: 10.1016/j.nbd.2006.02.006.

22. Midwood KS, Hussenet T, Langlois B, Orend G (2011): Advances in tenascin-C biology. *Cell Mol Life Sci*. . doi: 10.1007/s00018-011-0783-6.

23. Chiquet-Ehrismann R (2004): Tenascins. *Int J Biochem Cell Biol*. . doi: 10.1016/j.biocel.2003.12.002.

24. Tremble P, Chiquet-Ehrismann R, Werb Z (1994): The extracellular matrix ligands fibronectin and tenascin collaborate in regulating collagenase gene expression in fibroblasts. *Mol Biol Cell*. .

25. Duffy MJ (2001): Carcinoembryonic antigen as a marker for colorectal cancer: is it clinically useful? *Clin Chem*. .

26. Cooper ME, Vranes D, Youssef S, Stacker SA, Cox AJ, Rizkalla B, *et al.* (1999): Increased renal expression of vascular endothelial growth factor (VEGF) and its receptor VEGFR-2 in experimental diabetes. *Diabetes*. . doi: 10.2337/diabetes.48.11.2229.

27. Muffat J, Walker DW (2010): Apolipoprotein D: An overview of its role in aging and age-related diseases. *Cell Cycle*. . doi: 10.4161/cc.9.2.10433.

28. Furuhashi M, Tuncman G, Görgün CZ, Makowski L, Atsumi G, Vaillancourt E, *et al.* (2007): Treatment of diabetes and atherosclerosis by inhibiting fatty-acid-binding protein aP2. *Nature*. . doi: 10.1038/nature05844.

29. Shum BOV, Mackay CR, Gorgun CZ, Frost MJ, Kumar RK, Hotamisligil GS, Rolph MS (2006): The adipocyte fatty acid-binding protein aP2 is required in allergic airway inflammation. *J Clin Invest*. . doi: 10.1172/JCI24767.

30. Batterham RL, Le Roux CW, Cohen MA, Park AJ, Ellis SM, Patterson M, *et al.* (2003): Pancreatic polypeptide reduces appetite and food intake in humans. *J Clin Endocrinol Metab*. . doi: 10.1210/jc.2003-030630.

31. Denorme F, De Meyer SF (2016): The VWF-GPIb axis in ischaemic stroke: lessons from animal models. *Thromb Haemost*. . doi: 10.1160/th16-01-0036.

32. van der Laan SW, Fall T, Soumaré A, Teumer A, Sedaghat S, Baumert J, *et al.* (2016): Cystatin C and Cardiovascular Disease: A Mendelian Randomization Study. *J Am Coll Cardiol*. . doi: 10.1016/j.jacc.2016.05.092.

33. Mi W, Pawlik M, Sastre M, Jung SS, Radvinsky DS, Klein AM, *et al.* (2007): Cystatin C inhibits amyloid-β deposition in Alzheimer's disease mouse models. *Nat Genet*. . doi: 10.1038/ng.2007.29.

34. Heiss A, Eckert T, Aretz A, Richtering W, Van Dorp W, Schäfer C, Jahnen-Dechent W (2008): Hierarchical role of fetuin-A and acidic serum proteins in the formation and stabilization of calcium phosphate particles. *J Biol Chem*. . doi: 10.1074/jbc.M709938200.

35. Aggarwal S, Dabla PK, Arora S (2013): Prostasin: An Epithelial Sodium Channel Regulator. *J Biomarkers*. . doi: 10.1155/2013/179864.

36. Wang Y, Chen J, Chen L, Zheng P, Xu HB, Lu J, *et al.* (2014): Urinary peptidomics identifies potential biomarkers for major depressive disorder. *Psychiatry Res*. . doi: 10.1016/j.psychres.2014.02.029.
